# Perceived barriers to the management of foot health in patients with rheumatic conditions

**DOI:** 10.1186/s13047-015-0071-z

**Published:** 2015-04-14

**Authors:** Nina Lansdowne, Angela Brenton-Rule, Matthew Carroll, Keith Rome

**Affiliations:** AUT University, Health & Rehabilitation Research Institute and School of Podiatry, 90 Akoranga Drive, Auckland, 1142 New Zealand

**Keywords:** Rheumatoid arthritis, Gout, Rheumatic conditions, Multidisciplinary management, Foot, Podiatrists

## Abstract

**Background:**

Rheumatic conditions can have a significant impact on the feet and requires effective management. Podiatric involvement in the management of rheumatic conditions has previously been found to be inadequate in a hospital-setting and no study has examined current trends across New Zealand. The aim was to evaluate the perceived barriers of New Zealand podiatrists in the management of rheumatic conditions.

**Methods:**

A cross-sectional observational design using a web-based survey. The self-administered survey, comprising of thirteen questions, was made available to podiatrists currently practicing in New Zealand.

**Results:**

Fifty-six podiatrists responded and the results demonstrated poor integration of podiatrists into multidisciplinary teams caring for patients with arthritic conditions in New Zealand. Dedicated clinical sessions were seldom offered (16%) and few podiatrists reported being part of an established multidisciplinary team (16%). A poor uptake of clinical guidelines was reported (27%) with limited use of patient reported outcome measures (39%). The majority of podiatrists expressed an interest in professional development for the podiatric management of arthritic conditions (95%). All surveyed podiatrists (100%) agreed that there should be nationally developed clinical guidelines for foot care relating to arthritis.

**Conclusions:**

The results suggest that there are barriers in the involvement of podiatrists in the management of people with rheumatic conditions in New Zealand. Future studies may provide an in-depth exploration into these findings to identify and provide solutions to overcome potential barriers.

**Electronic supplementary material:**

The online version of this article (doi:10.1186/s13047-015-0071-z) contains supplementary material, which is available to authorized users.

## Background

Rheumatic conditions is a broad term used to describe a range of disorders of the joints and connective tissues including rheumatoid arthritis (RA), gout, systemic sclerosis, psoriatic arthropathy and systemic lupus erythematosus. These diseases are a leading cause of disability in adults [1,2] and a significant burden on the public healthcare system, both in New Zealand [3] and overseas [4]. In 2010, the prevalence of people aged 15 and over with arthritis was 15% in New Zealand [5]. New Zealand has both a public and private healthcare system but despite the notable burden that rheumatic conditions pose on the public healthcare system in New Zealand [3], there is currently a lack of podiatric integration within rheumatology services and an unmet need for podiatric foot care for patients with rheumatic conditions in NZ [6].

The literature is currently dominated by studies focusing predominantly on people with RA. The provision of foot care and education by the podiatrist is necessary as the feet often present as the initial site of involvement in RA. This can lead to foot pain, impairment and functional disability [6]. The podiatrist’s role involves the examination, diagnosis, management and education of foot and lower limb disorders [7]. Regular examination, education and the use of appropriate patient reported outcome measures allows for the monitoring of foot health status and changes, and the effectiveness of foot health interventions [7].

Severe physical, psychological and social consequences can be associated with rheumatic conditions. Therefore, a multidisciplinary management approach has been reported to address all aspects of the complex and variable needs of patients living with rheumatic conditions [8]. A multidisciplinary management approach, when effectively coordinated, has the potential to improve health outcomes, minimize waste and service duplication, and provide more accessible and timely care [9]. The investigation of the effectiveness of a multidisciplinary management approach has seen positive outcomes compared to a rheumatologist-centred approach [10].

Previous studies have reported an increasing request for specialist foot care services, with a strong support for the integration of podiatrists into the multidisciplinary management team for RA and other rheumatic conditions [6-13]. Although guidelines have been published in the UK [13] and Australia [14] for the role of the podiatrist in the multidisciplinary team, there are no national guidelines in New Zealand. While it may be inferred that podiatric management of patients with rheumatic conditions in New Zealand is similar to overseas practice, this area currently remains unknown. Therefore, the aim was to evaluate the perceived barriers to the management of foot health in patients with rheumatic conditions.

## Methods

The study was a cross-sectional observational design using a web-based survey. The survey was anonymous, self-administered and comprised of thirteen questions. Participants were recruited from Podiatry New Zealand (http://www.podiatry.org.nz/) and the Australasian Podiatric Rheumatology Special Interest Group (http://www.aprsig.co.nz/), using purposive sampling. Inclusion criteria were podiatrists that held a current New Zealand registration and excluded podiatrists currently practicing outside New Zealand. An overview of the study was displayed on the homepage of the PNZ and APRSIG websites with a hyperlink to the survey and a consent form. The reported response rate for web-based surveys is 30% [14]. Ethical approval was obtained from the Auckland University of Technology Ethics Committee (AUTEC).

The online software, Survey Monkey® (http://www.surveymonkey.com) was used. This software allows users to self-create surveys and is easy to use with a large set of features. Online surveys have the advantages of time efficiency, reduced cost, automated data collection and an ability to overcome distance barriers in participant data collection [15]. The survey questions were modified from a previous study conducted in Australia [13]. A mixture of dichotomous and nominal-polytomous close-ended questions were used, which have been shown to yield a higher percentage of answers, less missing data, and more adequate answers in online surveys [16]. The survey concluded with one ranking scale question. To ensure face and content validity the survey was piloted tested with local podiatrists specialising in rheumatology and by three international experts in the field of podiatric rheumatology. We also attempted to reduce both response and non-response bias by undertaking a number of pilot studies that ensured we used clear language, chose words and phrases with care, avoiding leading questions, providing the appropriate amount of options, reducing the number of questions to 13 and keeping the style of the survey to a minimum.

Questions 1–4 sought to obtain background demographic information on participants regarding sex, location of practice, years of registration and highest level of education (Additional file 1). To give insight into the current involvement of New Zealand podiatrists in the management of patients with rheumatic conditions, questions 5, 6, 7, 10 and 13 enquired into which rheumatic conditions they encountered in their practice, which professions were referring into their practice, whether any dedicated clinical sessions for rheumatic conditions were being offered, whether podiatrists are currently a part of a multidisciplinary management team, and their confidence in managing patients with rheumatic conditions.

To give insight into the use of provisions for the management of rheumatic conditions, questions 8, 9 and 11 enquired into the use of guidelines and outcome measures in practice. Question 12 enquired into the podiatrist’s opinion on further education, enabling insight into future research directions. Data analysis was conducted using Statistical Package for Social Science (SPSS) (version 20, SPSS Inc., Chicago, IL) with the primary analysis being descriptive statistics.

## Results

Fifty-six podiatrists participated in the study (response rate of 30%). Participants were practicing in 11 New Zealand regions, with the majority of respondents (n = 52, 93%) practicing in the North Island cities of Auckland and Wellington. There were a larger proportion of women (n = 34, 61%) than men (n = 22, 39%). The majority of participants had been registered for more than ten years (n = 20, 36%) or under one year (n = 16, 29%). A further 18% (n = 10) of respondents being registered for one to five years and 16% (n = 9) for six to ten years. Most respondents were qualified with a current Bachelor’s degree (n = 45, 80%). Six respondents (11%) held a diploma in podiatry and five (9%) held a Bachelor’s degree with Honours. Few participants held a Master’s degree (n = 4, 6%) or a Doctorate degree (n = 1, 2%).

**Figure 1:**
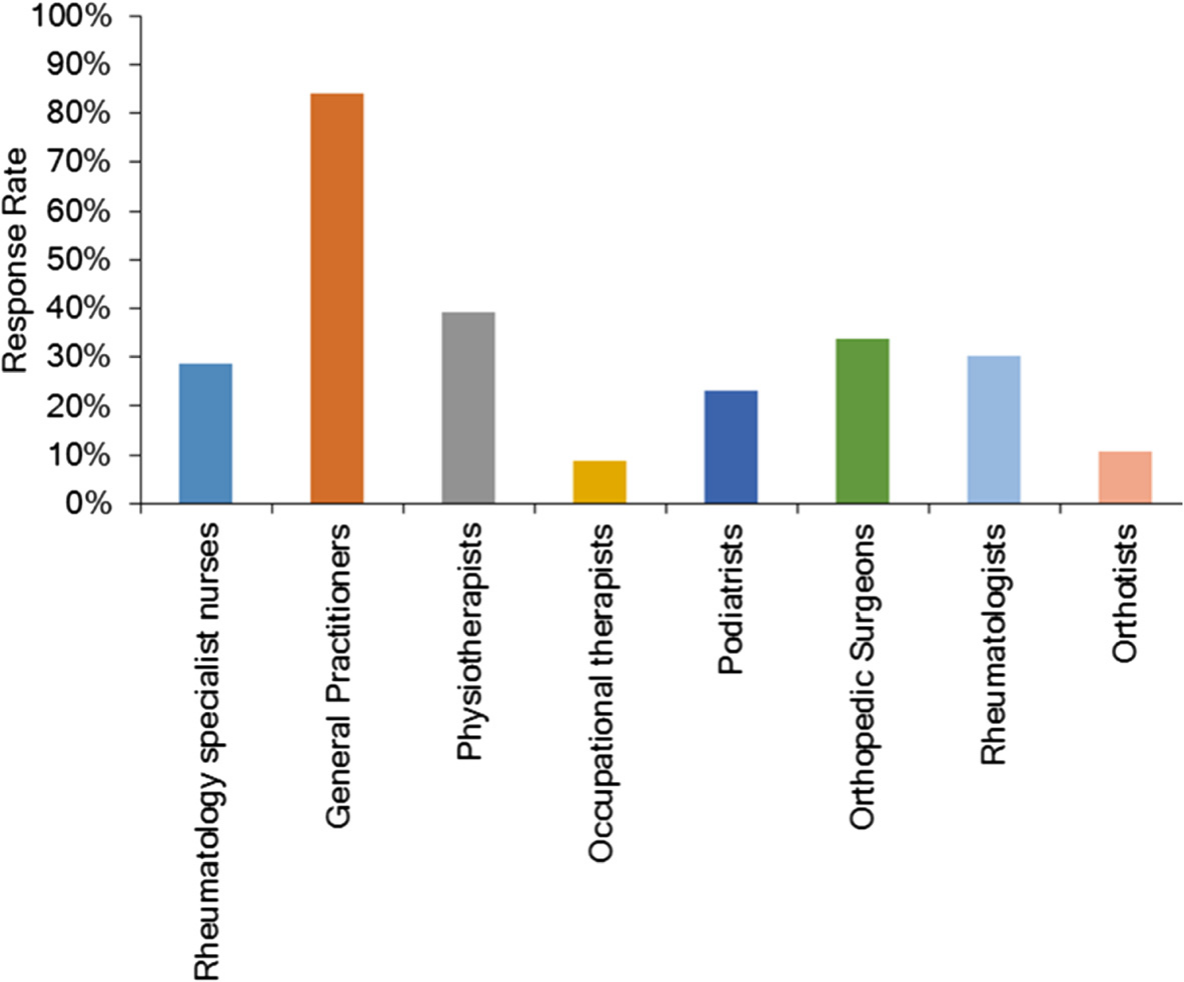
Bar graphs indicating which health professionals reportedly refer people with rheumatic conditions to Podiatry.

**Figure 2:**
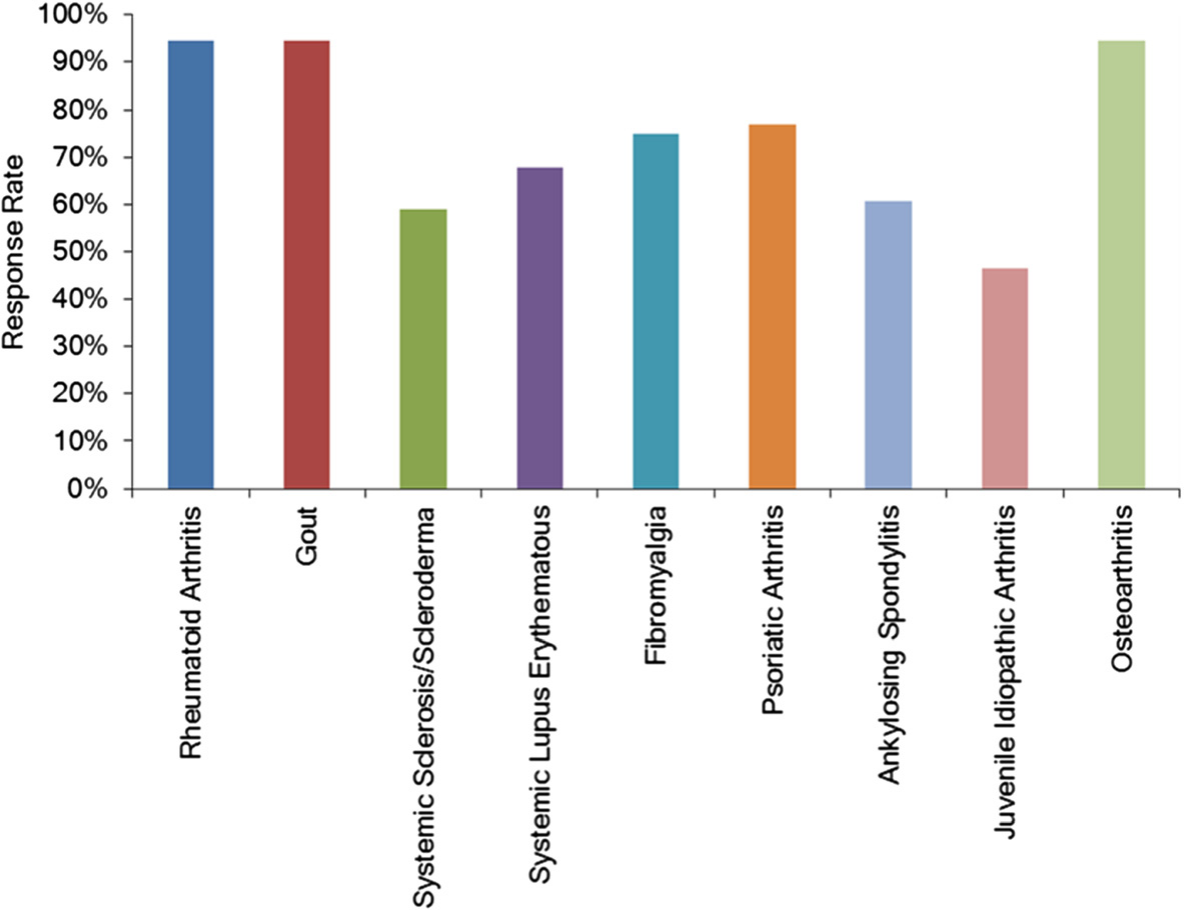
Bar graphs indicating which rheumatic conditions are reportedly seen within podiatric practice in New Zealand.

Eighty-four percent of participants (n = 45) indicated that general practitioners (GPs) refer people with rheumatic conditions into their clinic, making GPs the most common source of referral (Figure 1). The majority of participants (n = 53, 95%) reported managing people with RA, OA and gout (Figure 2). Most respondents did not offer clinical sessions specifically for people with rheumatic conditions (n = 45, 80%) and were not part of an established multidisciplinary team managing patients with rheumatic conditions (n = 45, 80%). Those respondents who were part of a multidisciplinary team (n = 9, 16%) indicated other team members to include orthotists, clinical nurse specialists, rheumatologists, researchers, GPs, other podiatrists, and orthopaedic surgeons. Multidisciplinary teams were identified as being located in a university-based podiatric rheumatology clinic and a hospital-based high-risk foot clinic.

**Figure 3:**
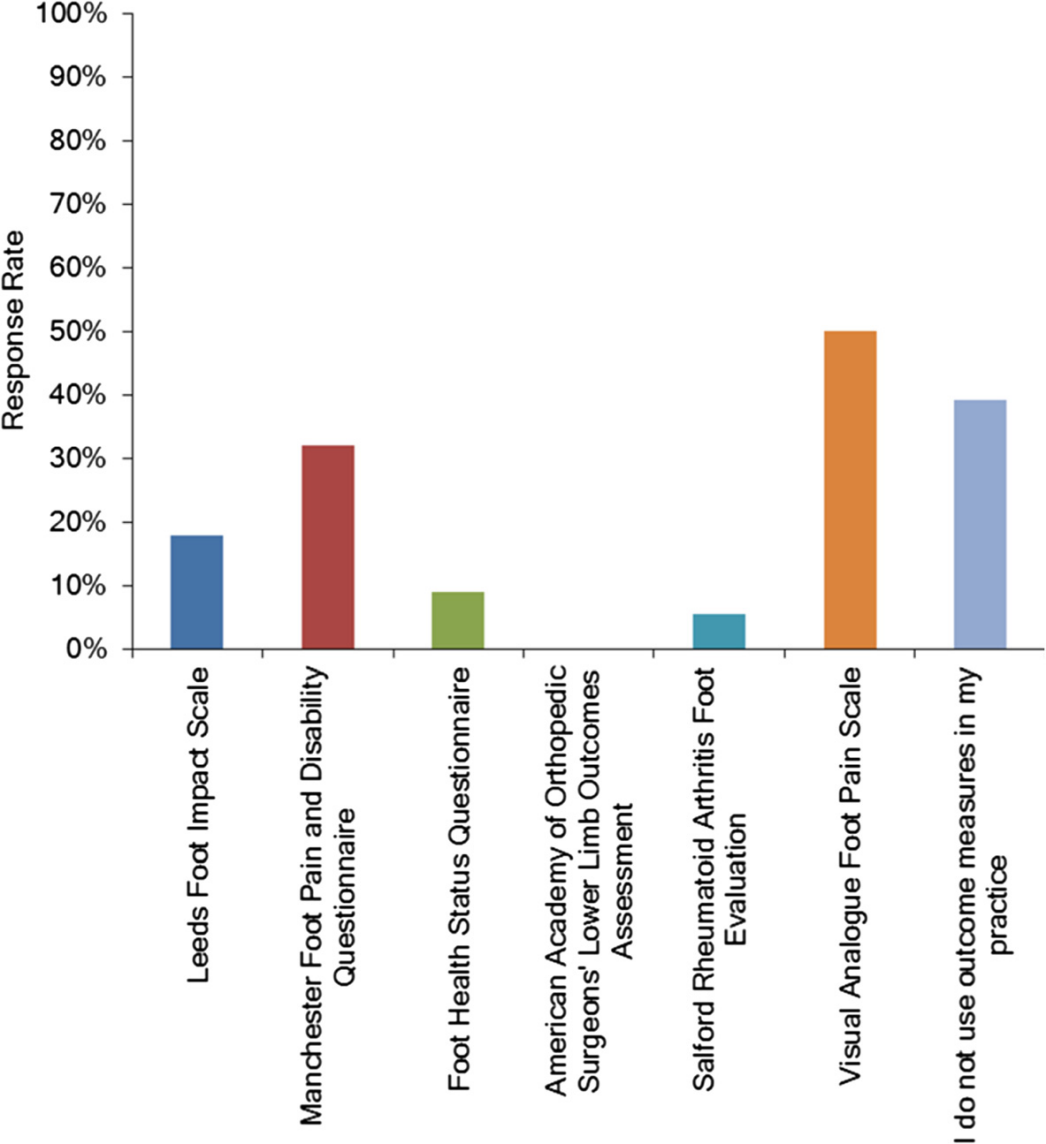
Bar graphs indicating the use of outcome measures when managing foot health in people with rheumatic conditions.

Just over one quarter of surveyed podiatrists indicated that they use clinical practice guidelines or protocols when managing people with rheumatic conditions (n = 15, 27%). A visual analogue foot pain scale was the most common outcome measure, used by 50% (n = 28) of respondents, with 39% (n = 22) of respondents indicating that they did not use any outcome measures in their practice. A low usage of other generic or specific foot outcome measures was reported (Figure 3). All participants responded positively when asked if there should be locally developed provisional guidelines for the podiatric management of patients with rheumatic conditions in New Zealand. Over 70% (n = 39) indicated that they would use them in their practice and the remaining participants indicated that they would read over them.

We found a large range of confidence in providing podiatric care for people with rheumatic conditions, ranging from feeling very confident to not feeling confident at all. A greater proportion of respondents felt somewhat confident in providing podiatric care for people with RA (n = 39, 70%), osteoarthritis (n = 36, 66%) and gout (n = 33, 59%). Almost all respondents (n = 53, 95%) indicated that they believe there should be more postgraduate opportunities for professional development in podiatric management of rheumatic conditions.

## Discussion

The findings of the study demonstrated that the majority of podiatrists were not involved in a multidisciplinary team managing people with rheumatic conditions. As a result, timely access to podiatric care may be hindered or the opportunity for foot care, for individuals with rheumatic conditions, may be missed. This is of significance as previous research has demonstrated that offering a podiatric service to individuals with rheumatic conditions can result in significant improvements in foot pain and disability [9].

With podiatry being a comparatively recently emerging profession, it may be that other health professionals are unaware of the complete role of the podiatrist and presume that all foot care needs can be fulfilled by other practitioners such as nurses and physiotherapists [17]. Further, it remains unknown how many surveyed podiatrists had actively sought to be part of a multidisciplinary team or recognize its value. Often the terms multidisciplinary versus interdisciplinary teams can incorrectly be used interchangeable. Multidisciplinary team approaches utilize the skills and experience of individuals from different disciplines, with each discipline approaching the patient from their own perspective. Interdisciplinary team approaches integrate separate discipline approaches into a single consultation [18]. The development of integrated podiatry care in New Zealand is reported to be progressing slowly with many unresolved challenges [9]. However, the current New Zealand trend toward integrated family health centres may provide opportunities to form effective multidisciplinary teams and partnerships, such as those reported between GPs and nurses [19,20].

The current study revealed that most New Zealand podiatrists are not offering clinical sessions specifically for patients with rheumatic conditions. Similar findings have been reported in the UK [8] and Australia [13] and emphasize the widely occurring inadequacy in foot health provision for people with rheumatic conditions. It is possible that the lack of specific clinical sessions offered by New Zealand podiatrists is reflective of either an underestimation of the burden of foot involvement in rheumatic conditions, particularly given New Zealand’s comparatively small population or there is a lack of skills from podiatrists in managing people with rheumatic conditions. Previous studies have reported patient dissatisfaction with the patient-doctor/rheumatologist relationship, alongside feeling that the seriousness of their foot problems are being overlooked [8,10]. Podiatrists are well placed to provide appropriate foot care and act as gatekeepers to other members of the multidisciplinary team, referring them in a timely manner when disease activity is observed [7,9,12,21]. The provision of rheumatology-specific clinics may facilitate this relationship.

We found that the majority of podiatrists received most referrals from GPs in comparison to other health professions. This referral tendency has also been found in Australia [13] and may reflect a good working relationship between podiatrists and GPs. The relatively low referral rates from other health professions may also be due to a lack of awareness of the podiatrist’s role by other health professionals. This lack of understanding of podiatry has also been documented from the patient’s perspective and has contributed to the inadequacies in foot care provision and delays in seeking appropriate foot care [8,11].

The current study demonstrated that only 27% of podiatrists were employing clinical guidelines in their practice when managing the foot. The implementation of clinical guidelines into healthcare practice has seen variable uptake despite evidence of improved patient outcomes [7,12,22]. It is unclear why the majority of New Zealand podiatrists surveyed are not using guidelines in their practice. However, it is becoming increasingly apparent that this is a widespread finding, with only 12% of Australian podiatrists adhering to guidelines [12] with similar findings in the UK [23]. A recent UK-based study also reported mixed impact of clinical guidelines from the podiatrist’s perspective, with some reporting their helpfulness in improving clinician and patient confidence in the ongoing care provided and others perceiving that patients would lose confidence in them if they knew they were using guidelines [21]. General barriers to use of clinical guidelines by physicians have also been previously reported including: lack of awareness, familiarity, agreement, self-efficacy, outcome-expectancy, inertia of previous practice and external barriers [24]. It is possible New Zealand podiatrists may feel that guidelines developed outside New Zealand are not readily applicable or they are unaware of any guidelines for people with rheumatic conditions, given the differing health care systems and contexts for which they were originally intended.

Despite the poor uptake of existing clinical guidelines, all podiatrists in the current study agreed that there should be national guidelines. This finding reflects positively on the willingness of New Zealand podiatrists to keep updated with best-practice recommendations. Further, in the current study, 70% of participants said they would use locally developed guideline. These findings are suggestive of a positive response if local guidelines for foot care in arthritis were developed, and warrant further exploration into this area. Further, the positive attitude reflected in the current survey may contribute to helping overcome the potential barriers to guideline use, including lack of time in clinical practice to read guidelines, and the existence of a large range of clinical guidelines with overlapping information [22].

We found inconsistencies in the use of outcome measures in clinical practice. Similar to clinical guidelines, outcome measures are an essential component of evidence-based practice and are of benefit at the population and the patient level [25,26]. Barriers to their implementation in palliative care include time management, education, practitioner motivation, finances, the specificity of the outcome measure and education [26]. With the advent of multidisciplinary teams involved in the care of patients with RA, outcome measures can be of benefit to the whole multidisciplinary team [27]. Although the current finding of 40% of podiatrists not using outcome measures in clinical practice is an important finding, this rate is significantly lower than the 71% of those that are not using outcome measures reported in a recent Australian study [13].

Examining the podiatrists’ confidence in patient management has found variable results between different arthritic conditions. Podiatrists surveyed in the UK have previously reported confidence in managing aspects of RA [13]. Other rheumatic conditions have not been examined, however, and the podiatrist’s confidence in overall management was reported to be lower than that of other health professionals, including physiotherapists and occupational therapists. This has clinical implications as foot problems can be missed if a practitioner’s confidence in assessing the feet in rheumatology is lacking.

We found that almost all podiatrists agreed that there should be more opportunities for professional development in arthritis. A recent UK study demonstrated graduate education in rheumatology to be of value to the podiatrist’s general career as well as helping maximize their personal and professional development [28]. Research focusing on GPs also showed increased confidence in managing a majority of musculoskeletal conditions with the provision of ongoing education opportunities [29]. Furthermore, it may also be beneficial to the podiatry profession if further education and training involved not only clinical skills, but also explored elements relating to patient satisfaction, which has been reported as a barrier to foot care delivery [7].

The study has limitations. The number of participants represents only 18% of the New Zealand podiatry workforce, since not all registered podiatrists in New Zealand are members of the New Zealand professional body, Podiatry New Zealand (PNZ) or the Australasian Podiatric Rheumatology Special Interest Group (ASPRIG). Therefore, limiting the generalisability of the results to the wider podiatry profession in New Zealand and world-wide [30,31]. While this research examines the podiatrist perspective and current involvement within a multidisciplinary team, future research is warranted to examine trends in the management of people with rheumatic conditions within an integrated care setting. An examination of the perspectives of other health professionals with regard to podiatrists in this setting would also be of value and may provide some direction toward achieving greater integration of podiatrists in multidisciplinary teams and integrated care settings in New Zealand.

## Conclusion

This study has provided the first insight into the perceived barriers of podiatric involvement in the management of rheumatic conditions, providing a foundation and direction for further exploration into this area. It seems that a large opportunity exists for New Zealand podiatrists and the podiatry profession to facilitate integration within a multidisciplinary team setting and develop referral pathways to enable the optimal management of the feet. With the need for further education opportunities recognized by all New Zealand podiatrists and their interest in having locally developed guidelines, now appears to be the time to direct resources into moving this area forward in the hopes of improving rheumatic conditions management for both the patient and clinician.

## Additional file

Additional file 1 Online podiatric survey.
