# Supplementary material for: Perceived barriers to the management of foot health in patients with rheumatic conditions
Source: J Foot Ankle Res. 2015 Apr 14;8:14. doi: 10.1186/s13047-015-0071-z (PMC4404570; doi:10.1186/s13047-015-0071-z)
Supplement: Additional file 1 — Online podiatric survey. [file 13047_2015_71_MOESM1_ESM.docx]

ONLINE PODIATRIC SURVEY

1. **What is your gender?**

Male

Female

2. **In which region of New Zealand are you currently practicing?**

Northland Nelson

Auckland Marlborough

Waikato West Coast

Bay of Plenty Canterbury

Gisborne Timaru/Oamaru

Hawkes Bay Otago

Taranaki Southland

Wanganui

Manawatu

Wairarapa

Wellington

3. **How many years have you been registered as a podiatrist?**

Under 1 year

1-5 years

6-10 years

More than 10 years

4. **What is your highest level of education with regard to podiatry and specialised areas of podiatry? Please select all that apply (If you have a higher level of education in another area, please also select ‘other’ below and specify)**

Diploma in Podiatry

Bachelor’s degree (or overseas equivalent)

Bachelor’s degree with honours

Master’s Degree

PhD

Other: please specify

5. **Which rheumatic conditions have you come across in your practice? Please select all that apply.**

Rheumatoid arthritis

Gout

Systemic sclerosis/scleroderma

Systemic Lupus Erythematous

Fibromyalgia

Psoriatic arthritis

Anylosing spondylitis

Juvenile idiopathic arthritis

Osteoarthritis

Other: please specify

**6. Which of the following health care professionals currently refer patients with rheumatic disease directly into your clinic? Please select all that apply.**

Rheumatology specialist nurses

General practitioners

Physiotherapists

Occupational therapists

Podiatrists

Orthopaedics Surgeons

Rheumatologists

Orthotists

Other: please specify

**7. Do you offer dedicated clinical sessions specifically for your patients who have rheumatic disease? (For example, do you run a clinic solely for patients with inflammatory arthritic conditions).**

Yes

No

**8. Do you use any formal guidelines or protocols for the podiatric management of patients with rheumatic disease?**

Yes

No

**9. Please select all of the following outcome measures that you use to manage patients with rheumatic disease in your practice.**

Leeds Foot Impact Scale

Manchester Foot Pain and Disability Questionnaire

Foot Health Status Questionnaire

American Academy of Orthopaedic Surgeons’ Lower Limb Outcomes Assessment

Salford Rheumatoid Arthritis Foot Evaluation

Visual Analogue Foot Pain Scale

I do not use outcome measures in my practice

Other: please specify

**10. Are you part of an established multidisciplinary team in managing patients with rheumatic disease? (I.e. you have been working together with other health professions to manage an individual with rheumatic disease, and feel comfortable contacting these members and discussing the particular case and its current management approaches)**

Yes

No

If yes, please briefly describe the structure of the multidisciplinary team

**11. Do you think there should be provisional guidelines for the podiatric management of rheumatic disease specific to New Zealand?**

Yes, I would likely employ them in my podiatric practice

Yes, but just to read over

No, there are sufficient provisions already for rheumatic disease management

No, provisions are not necessary in rheumatic disease management

**12. Do you believe there should be more opportunities for professional development (post-graduate) in podiatric rheumatic disease?**

Yes

No

**13. Rate your confidence in providing podiatric care for the following conditions:**

|  | Very Confident | Somewhat Confident | Neutral | Not very Confident | Not Confident |
| --- | --- | --- | --- | --- | --- |
| Rheumatoid arthritis |  |  |  |  |  |
| Gout |  |  |  |  |  |
| Systemic sclerosis/scleroderma |  |  |  |  |  |
| Systemic Lupus Erythematous |  |  |  |  |  |
| Fibromyalgia |  |  |  |  |  |
| Psoriatic arthritis |  |  |  |  |  |
| Anylosing spondylitis |  |  |  |  |  |
| Juvenile idiopathic arthritis |  |  |  |  |  |
| Osteoarthritis |  |  |  |  |  |
